# Interactions of Interaural Time and Level Differences in Spatial Hearing with Cochlear Implants

**DOI:** 10.1002/advs.202500918

**Published:** 2025-11-14

**Authors:** Sarah Buchholz, Susan Arndt, Jan W. H. Schnupp, Nicole Rosskothen‐Kuhl

**Affiliations:** ^1^ Neurobiological Research Laboratory Section for Experimental and Clinical Otology Department of Otorhinolaryngology Medical Center–University of Freiburg Faculty of Medicine Killianstr. 5 79106 Freiburg Germany; ^2^ Section for Experimental and Clinical Otology Department of Otorhinolaryngology Medical Center – University of Freiburg Faculty of Medicine 79106 Freiburg Germany; ^3^ Department of Neuroscience City University of Hong Kong Hong Kong SAR Sha Tin China; ^4^ Gerald Choa Neuroscience Institute Chinese University of Hong Kong Sha Tin Hong Kong SAR Sha Tin China; ^5^ Bernstein Center Freiburg and Faculty of Biology University of Freiburg 79104 Freiburg Germany; ^6^ Department of Otorhinolaryngology Head and Neck Surgery Chinese University of Hong Kong Hong Kong SAR Sha Tin China

**Keywords:** auditory neuroscience, auditory prostheses, cochlear implants

## Abstract

Normally hearing humans can localize sound sources quite accurately, with minimum audible angles as small as 1°. To achieve this, these auditory pathways combine information from multiple acoustic cues, including interaural time and interaural level differences (ITDs and ILDs). Patients relying on cochlear implants (CIs) to hear the world do not match normal performance. These deficits are most pronounced in patients with little or no hearing experience early in life, and they appear to result from impaired sensitivity to ITDs, but not to ILDs. However, little is known about how ITD and ILD sensitivities develop and interact in an early deafened auditory system shortly after CI implantation. neonatally deafened rats with bilateral CIs are fitted, and, providing informative ITDs and ILDs from stimulation onset, trained them to lateralize CI stimuli. These animals are exquisitely sensitive to both ILDs and ITDs of CI stimulus pulses, and combined information from both cues in a weighted sum. Importantly, ITDs are weighted heavily in the CI rats, such that only very modest ITDs pointing in one direction can confound quite large ILDs pointing in the opposite direction. This underlines the importance of informative ITDs for maximizing the potential for spatial hearing with CI devices.

## Graphical abstract

Differences in timing (Δ*T*) are weighted heavily compared to differences in loudness (Δ*L*) in binaural hearing with cochlear implants.

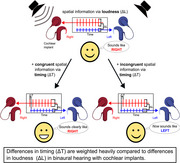

## 1 Introduction

Our ability to hear, to detect, identify, and also to localize sound sources, allows us to interact with and navigate through our acoustic environment. Spatial hearing relies on the brain's capacity to process binaural cues, including interaural time differences (ITDs) and interaural level differences (ILDs). Our brain can determine differences in both arrival time and sound amplitude of incoming sound at each ear with remarkable accuracy, and it uses both as cues for sound source direction. Importantly, in natural hearing, the physical acoustics of the head and outer ears ensures that ITDs and ILDs are tightly coupled, such that, for example, sounds arriving from the left will arrive slightly earlier, as well as being slightly louder, in the left ear. To achieve accurate and reliable estimates of sound source directions, the auditory system sub‐consciously combines the information from both cues. One consequence of this is that, when we deliver sounds over headphones with ITD and ILD cues that disagree, say, one pointing left, the other right, then we typically perceive the sound source at a “compromise location” somewhere in the middle. This phenomenon has been called “cue trading,” and it can be quantified in terms of the “time‐intensity trading ratio” (TITR), a psychophysical measure that indicates how many µs of ITD pointing in one direction are required to cancel out a 1 dB ILD pointing in the opposite direction.^[^ ^1^, ^2^, ^3^, ^4^, ^5^ ^]^ The TITR thus quantifies the relative strength of ITD and ILD cues on overall spatial perception in µs dB^−1^. TITRs reported in the literature for acoustic stimulation range from approximately 2.5 to 185 µs dB^−1^.^[^ ^3^, ^4^, ^5^, ^6^, ^7^, ^8^ ^]^ This broad range is attributable to the fact that in subjects with normal hearing, TITRs are somewhat stimulus dependent, with both sound frequency^[^ ^3^, ^9^, ^10^ ^]^ and sound intensity^[^ ^5^ ^]^ influencing TITR values. TITRs are lower for lower frequencies, which is probably adaptive, given that low frequency, long wavelength sounds create much smaller ILDs than high frequency ones. At these frequencies, large changes in source direction would produce only small and hard to detect changes in ILD, and it is therefore probably advantageous for the auditory system to place greater weight on ITD information for these low frequencies. Although the determinants of the TITR for a given stimulus type remain unclear, these ratios can nevertheless be seen as part of a “recipe” by which the auditory system combines timing and intensity cues to optimize spatial localization. The neural mechanisms underlying this cue integration are poorly understood, and the relative roles of evolution versus individual sensory experience in shaping these circuits remain unknown. This also limits our ability to predict how the auditory system adapts to situations such as technologically assisted hearing, where independent stimulation of each ear by bilateral cochlear implants (CIs) or headphones can lead to a prolonged uncoupling of ITDs and ILDs.

In this context, cochlear implants are of particular interest. All contemporary clinical devices will place separate processors at each of the listeners ears, and with very few exceptions, these will produce fixed rate pulsatile electric stimulation, with a pulse timing that is entirely independent of the incoming sounds. Consequently, they will produce random pulse timing ITDs which are entirely uninformative about sound source direction, as well as entirely uncoupled from ILDs.^[^ ^11^ ^]^ Interestingly, patients fitted with bilateral CIs typically exhibit good sensitivity to ILDs,^[^ ^12^, ^13^, ^14^ ^]^ but their ITD sensitivity tends to be poor.^[^ ^15^, ^16^ ^]^ This poor ITD sensitivity is particularly striking against the background of reports that rats, which were implanted with bilateral CIs but were never exposed to prolonged stimulation with uninformative pulse timing ITDs, do in fact show excellent sensitivity to these binaural cues, both behaviorally^[^ ^17^, ^18^, ^19^ ^]^ and electrophysiologically.^[^ ^20^ ^]^ What causes the poor ITD sensitivity in bilateral CI patients remains somewhat controversial, and several researchers have favored the idea that a failure of normal binaural development occasioned by early onset hearing loss may be at least partly to blame. So far, less attention has been paid to the possibility that the randomized pulse timing ITDs delivered by the great majority of electrode channels in clinical devices could be grossly disruptive if the auditory pathway is initially set up to combine useful information gleaned from ILDs with uninformative and misleading random number pulse timing ITDs. We therefore suspect that, rather than an absence of normal experience, it may be the presence of a sustained experience of uninformative pulse timing ITDs that ultimately leads to poor ITD sensitivity. Presumably, the auditory system tries to reduce the confounding influence of this cue that has been permanently rendered unreliable by the design choices of CI stimulation strategies.^[^ ^11^ ^]^

Of course, this “ITD desensitization theory” presupposes that, prior to prolonged stimulation with random pulse timing ITDs, firstly that the auditory pathway of CI patients had more or less normal sensitivity to ITDs, and secondly that it would combine ITDs and ILDs in a more or less normal manner, such that the two cues can synergize if they are congruent, but they can also confound each other when they conflict. Furthermore, this would be true even in the case of patients born with profound hearing loss, who had no opportunity to benefit from normal sensory experience that could have guided the development or refinement of their binaural hearing. CI recipients who have not yet been subjected to prolonged stimulation with clinical processors should therefore lateralize binaural stimuli more strongly when ITDs and ILDs covary, hear them closer to the midline if ITDs and ILDs conflict, and exhibit relatively small TITR values, effectively signaling that their auditory system expects ITDs to make a valuable contribution to overall localization judgments. Unfortunately testing these predictions in CI patient volunteers is extremely difficult, and consequently we have set out to test them here in our previously established animal model.

For this purpose, we work with neonatally deafened, adult bilaterally implanted CI rats, which gives us full control over stimulation parameters as well as hearing experience. These animals are relatively easy to train, typically within a couple of weeks, in stimulus lateralization tasks, and consistently exhibit excellent sensitivity to both binaural cues, ITD and ILD with only modest amounts of training, after activation of the CIs.^[^ ^17^, ^18^, ^20^, ^21^ ^]^ This has now made it possible for us to investigate how ITD and ILD cues interact in a CI stimulated, mammalian auditory pathway, which has not been conditioned and perhaps ITD‐desensitized, by lengthy exposure to clinical CI processors.

In this study, we investigated how our CI rats combined ITD and ILD cue information to make stimulus lateralization judgments by observing their responses to a set of different ITD/ILD combinations over many hundreds of trials. Our results clearly indicate that in these neonatally deafened CI rats, which had no experience of acoustic hearing and only relatively modest amounts of experience with CI hearing, ITDs and ILDs combine to inform sound source location judgments much as would be expected in natural hearing, with congruent cues reinforcing each other to provide a more strongly lateralized percept, and conflicting cues being traded off against each other to bring the percept closer to the midline. A probit regression model which assumed an additive effect of ITD and ILD cues gave a good fit to the behavioral data, and allowed us to calculate precise estimates of the animals TITRs. The low TITR values we observed not only indicate that ITDs are actually very heavily weighted in sound direction judgments made by an auditory system that has not undergone months of conditioning with randomized pulse timing ITDs, they also indicate that the randomized ITDs delivered by typical clinical CI stimulation may indeed have the potential to powerfully perturb ILD based sound source lateralization judgments unless the system adapts, for example by becoming much less sensitive to ITD.

## 2 Results

**Figure 1:**
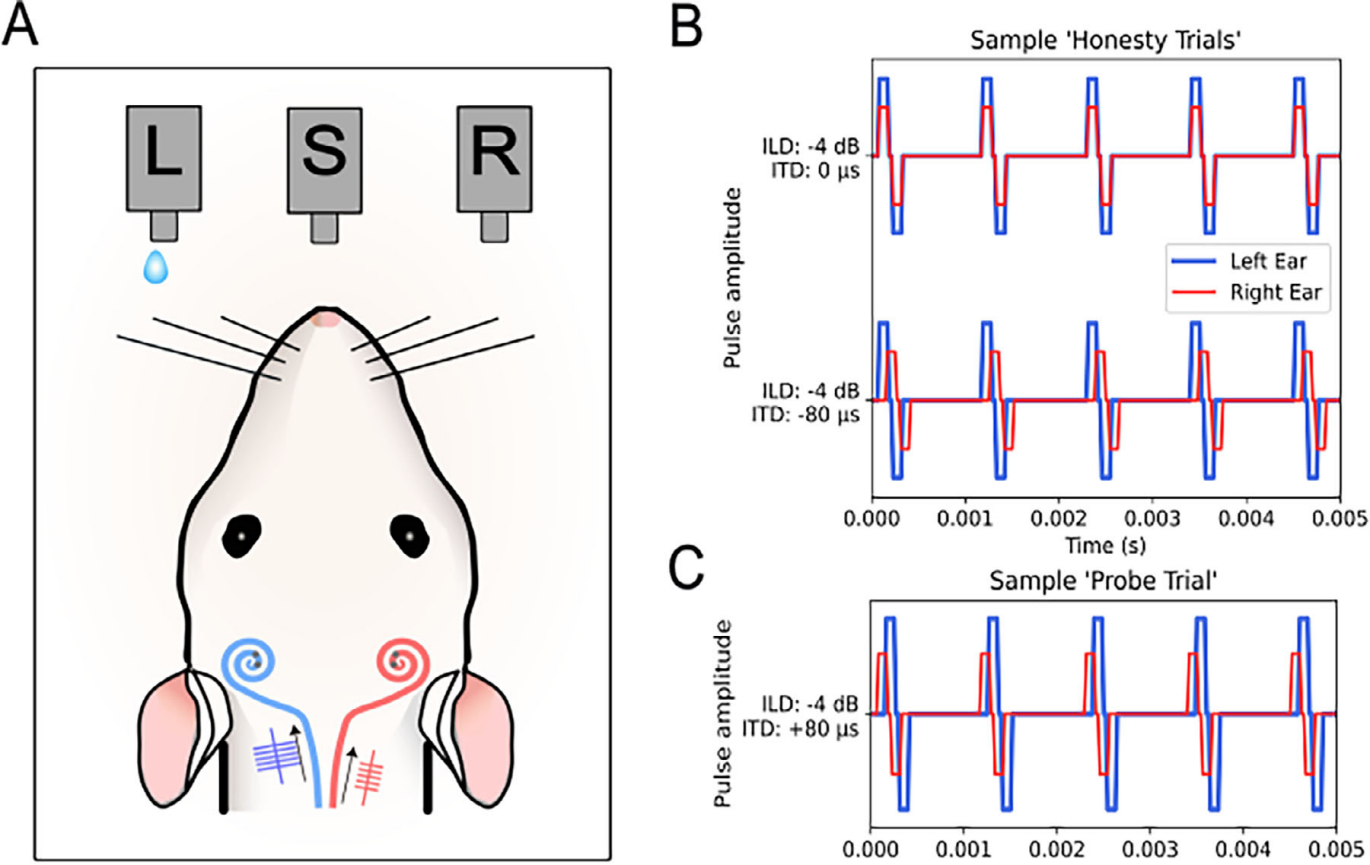
A) Schematic of a neonatally deafened, cochlear implanted (CI)‐supplied rat performing a two‐alternative forced choice sound lateralization task in our custom‐build behavioral setup. The rat needs to trigger the center spout (S) to receive a stimulus and then respond either on the left (L) or right (R) spout, accordingly. In this example, the stimulus arrives earlier and with higher intensity on the left CI, and only a response on the left water spout (L) would be rewarded. Two sample “honesty trials“ are displayed in (B). A binaural electric stimulus pulse train with an ILD of ‐4 dB and an ITD of 0 µs is shown in the upper panel, a pulse train with an ILD of ‐4 dB and an ITD of ‐80 µs is shown in the lower panel. In both examples the rat would need to respond on the left spout to receive drinking water as positive reinforcement. An incorrect response would lead to a timeout. C) Example of a “probe trial“, here the binaural electric stimulus pulse train contains an ILD of ‐4 dB, pointing to the left, and an ITD of +80 µs, pointing to the right. During probe trials the rat had a free choice and would be rewarded irrespective of the side they chose.

All nine neonatally deafened (ND) CI rats learned to lateralize binaural electric stimuli in a 2‐alternative‐forced choice (2AFC) sound lateralization task using 900 pps stimulation rate. **Figure** **1**A represents the behavioral testing setup. The ITD and ILD of the pulse train informed the rat's choice of which one of two response spouts to lick to receive water as a reward. To determine the rats’ joint ITD and ILD sensitivity, we tested their responses to combinations of ITDs {0, ±60, ±80} µs and ILDs {0, ±1, ±4} dB. All possible combinations were tested, except (0 µs, 0 dB). Stimuli with congruent ITDs and ILDs are referred to as “honesty trials”, in which the animals had to respond on the correct side to receive a reward. Trials with conflicting ITDs and ILDs are referred to as “probe trials”, in which the animals were free to respond according to their individual perception. Figure 1B,C shows examples of honesty and a probe trial stimuli. To avoid biasing the animals’ TITRs through reinforcement during testing, all probe trials were rewarded irrespective of the animal's choice. To discourage animals from a strategy of random guessing without listening carefully to the stimuli, we randomly interleaved honesty and probe trials, with honesty trials outnumbering probe trials by approximately 4:1. See the Experimental Section for further details.

**Figure 2:**
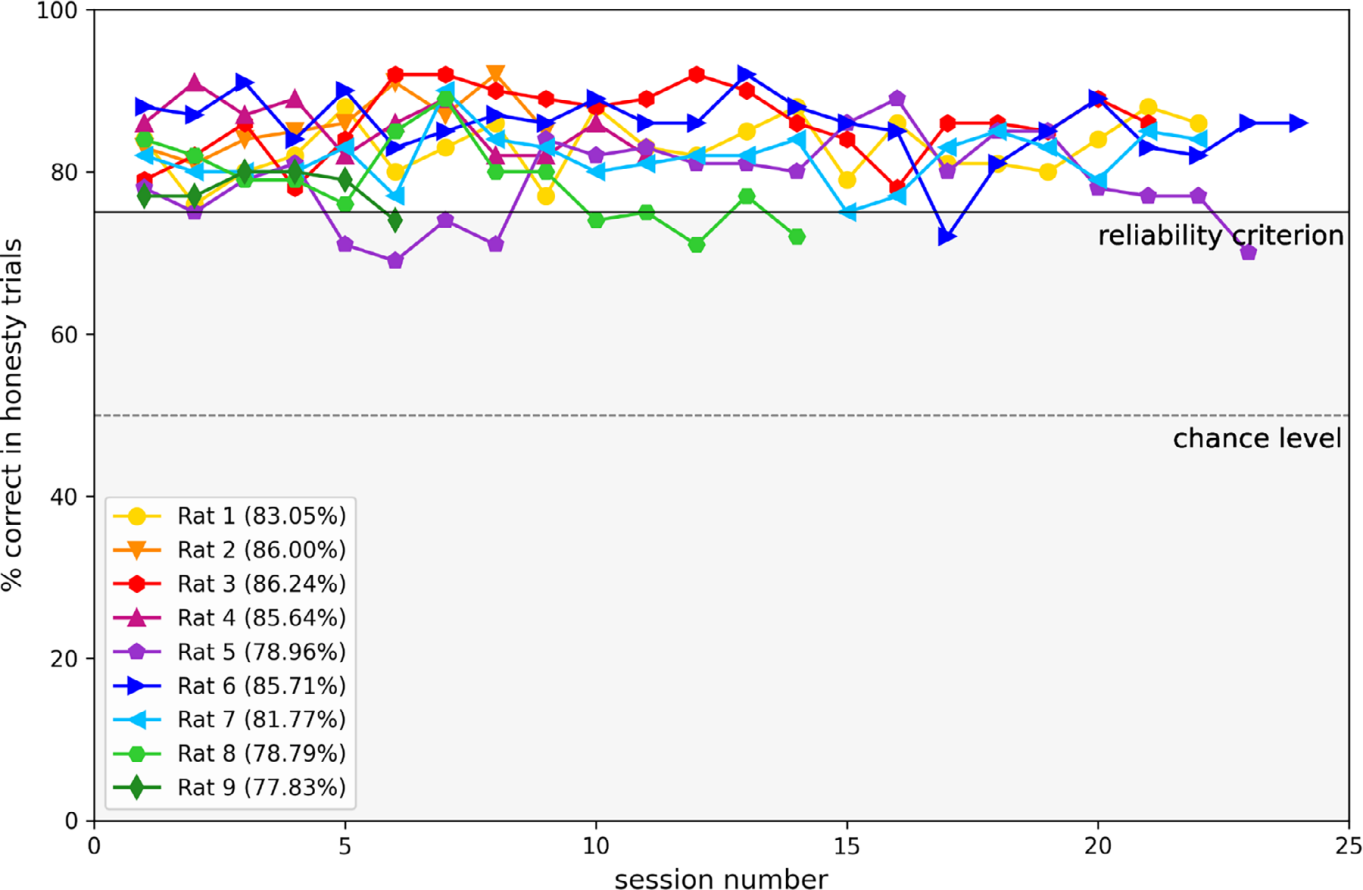
Performance of individual animals indicated by color for “honesty trials“ with congruent binaural cue information, mean performance across all sessions given in parentheses. A performance of 50% corresponds to chance level (indicated by the lower gray dashed line). The reliability criterion was set at 75% performance, as values at or above this threshold indicate consistent responding. Only data meeting this criterion were included in the analysis.

The honesty trials ensured that the rats performed the task reliably, and only sessions in which the animal's performance reached or exceeded 75% correct responses to honesty trials were included in further analysis. **Figure** **2** shows the performance of all CI animals across the individual testing sessions. Considering some fluctuations in performance are to be expected given that each animal's motivation, focus and alertness cannot be expected to be constant throughout the approximately three weeks of testing for this study, we nevertheless observed that the performance was quite stable over time, always well above chance, and only rarely dipping below our 75% reliability criterion.

**Figure 3:**
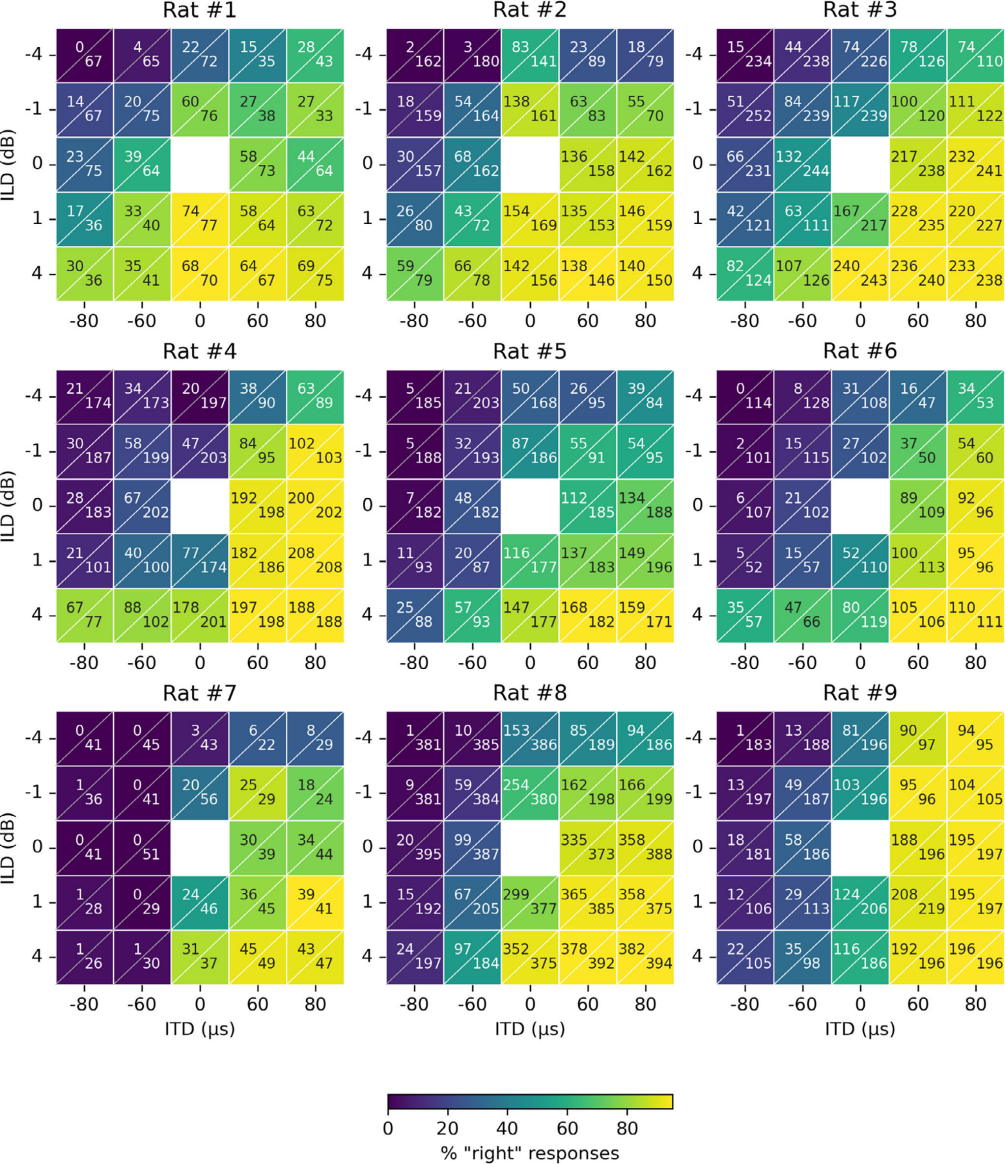
Heatmaps showing the proportion of responses to the right‐hand side for the different ITD (columns) and ILD (rows) combinations for nine neonatally deafened cochlear implanted rats (headings). Each cell shows two numbers, the upper left number indicates the number of responses to the right‐hand side and the lower, right number gives the total number of presented trials, resulting in the proportion of responses to the right hand side as indicated by the colorbar at the bottom.

In total we collected between six and 23 sessions reaching the performance criterion for each animal, which comprised between 849 and 7098 trials. The lateralization judgments from these trials, as a function of stimulus ITD and ILD, are summarized as heatmaps in **Figure** **3**. In each panel, responses to different ILDs are arranged bottom to top, while ITDs are arranged left to right. In each cell two numbers are shown. The top left indicates the number of trials for which the rat responded on the right reward spout, while the bottom right number reports the total number of trials performed for the specific parameter combination. The corresponding percentage of “right” responses is also represented by the color of each cell: the lighter the color, the lower the percentage of responses to the right hand side and the darker the color, the higher the percentage of responses to the right hand side (see color bar bottom of Figure 3). This allows us to visualize the effects of ILD and ITD as color gradients running top‐to‐bottom or left‐to‐right, respectively. Most animals show a very clear gradient in the diagonal direction from top left to bottom right, as would be expected if the animals are sensitive to both ITD and ILD, and these two cues have more or less additive effects on the animals’ lateralization judgments. For some animals (e.g., rat 7), the left‐to‐right color gradient is a lot more obvious than the top‐to‐bottom gradient, indicating that these animals were a lot more influenced by ITD than ILD for the parameter ranges tested.

**Figure 4:**
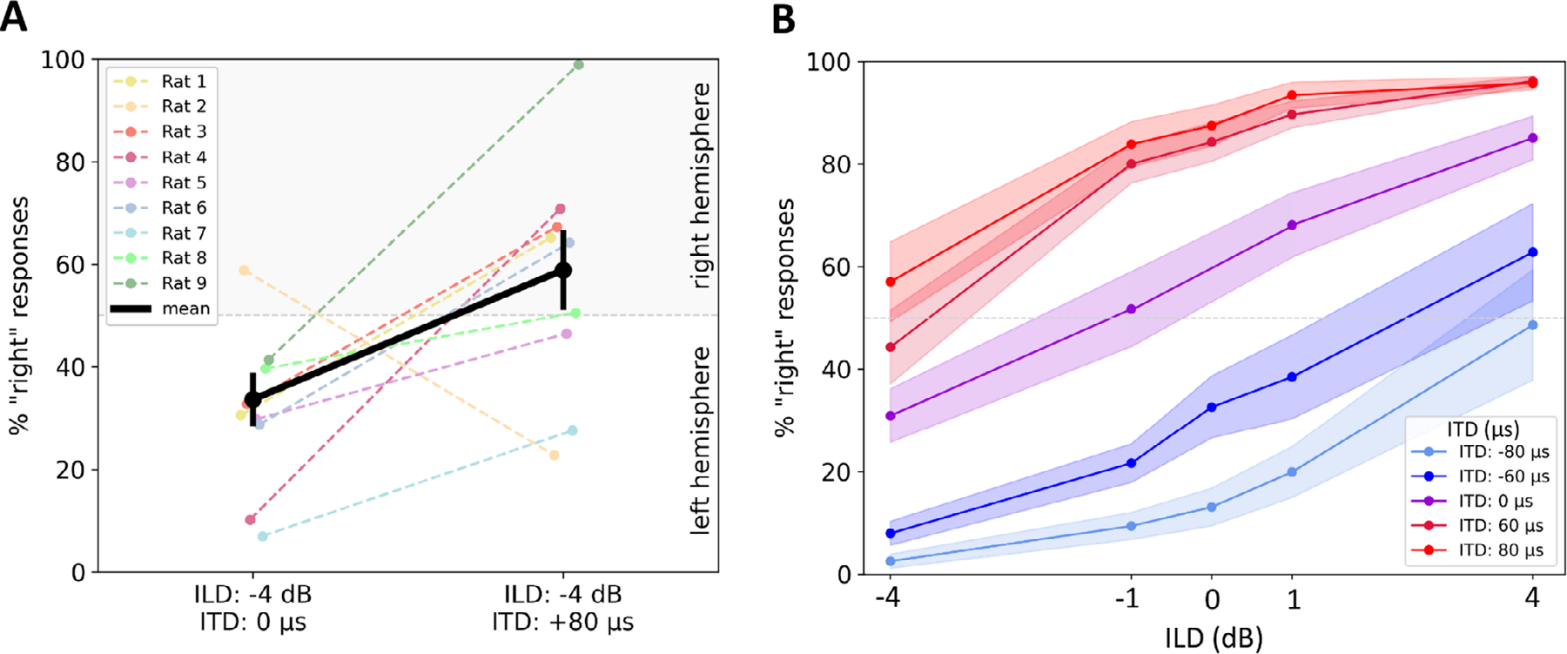
Percentage of responses to the right hand side of all nine CI rats for the different binaural cue combinations. The y‐axis gives the percentage of responses to the right hand side. Since left or right were the only possible choices, values below 50% indicate a prevalence of responses on the left. A) Shows the individual data of the nine rats, as indicated by colors for the two cue combinations ILD ‐4 dB, ITD 0 µs and ILD ‐4 dB, ITD +80 µs. The mean across all animals is displayed in black with error bars showing the standard error of mean. B) Shows the effect of changing ILD on lateralization judgments, averaged across all rats, for different ITDs (indicated by the colored curves), with the corresponding ILD values on the x‐axis.

As an example of how the change of one binaural cue affects the overall resulting lateralization decisions, **Figure** **4**A shows the percentages of responses to the right hand side for two binaural cue combinations. When the combination of ILD = ‐4 dB (left leading) and ITD = 0 µs (frontal) was presented, the animals responded on the right side in on average 34% of the trials. Adding an incongruent ITD cue of +80 µs (right leading) strongly affected the lateralization decisions of the rats, resulting in a preference of the right hemisphere in 59% of the trials. In order to obtain a more general overview of how strongly and systematically ILD psychometric functions can be shifted by even just quite small changes in ITD, Figure 4B shows the mean percentage of right responses across all rats (± SEM as shaded areas) for the different cue combinations. The x‐axis represents stimulus ILD, the y‐axis the observed percentage of “right” lateralization decisions. The purple curve represents the ILD psychometric function at ITD = 0 µs. The two red curves correspond to right‐leading ITDs and are shifted upward, reflecting an increased proportion of rightward responses. The blue curves correspond to left‐leading ITDs and are shifted downward.

**Figure 5:**
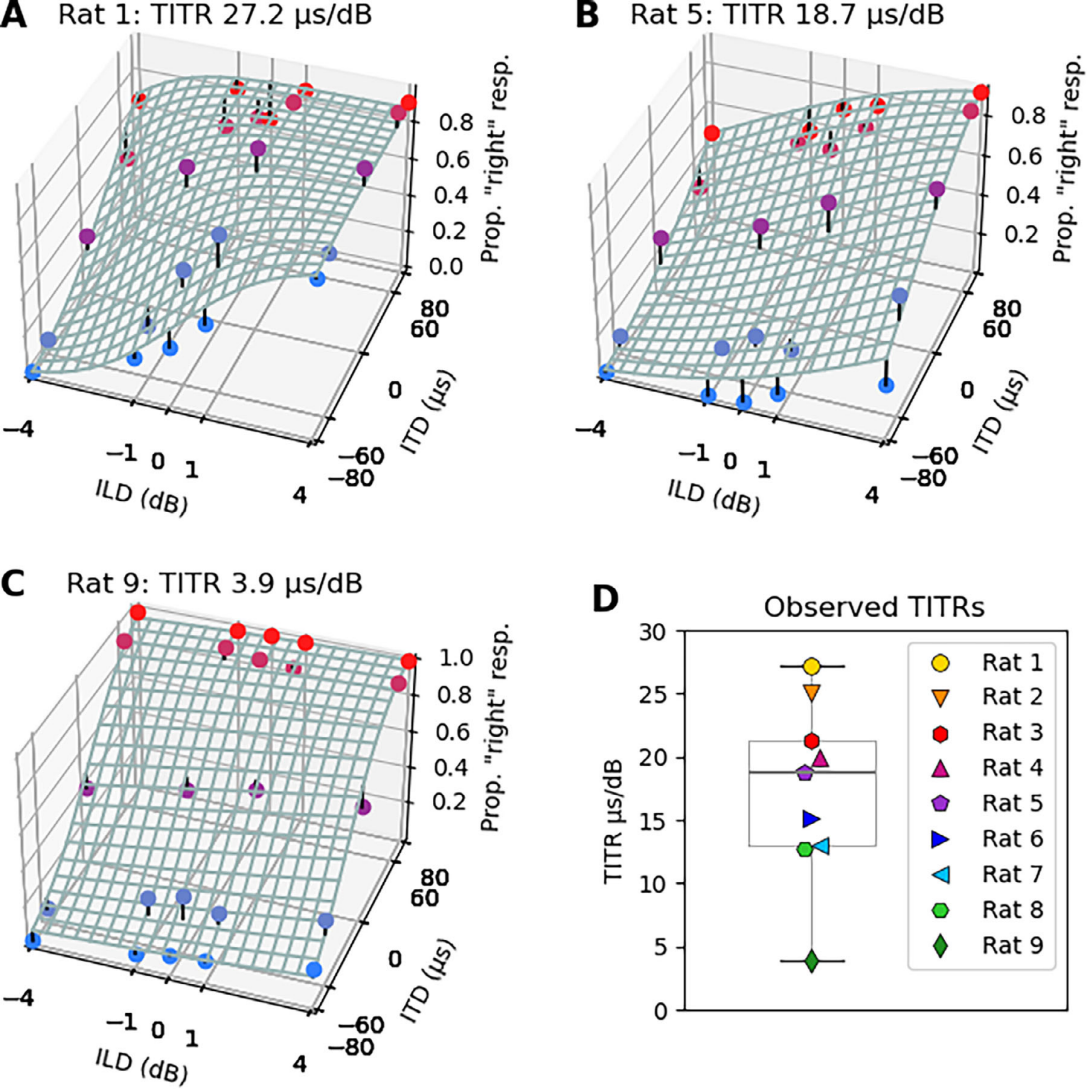
Sample 3D joint ITD and ILD psychometric functions fitted to the raw data of three sample CI rats (A–C). X‐axis: ILD values in dB with negative values representing higher intensity on the left ear. Z‐axis: ITD values in µs with negative values representing ITDs arriving earlier on the left ear. Y‐axis: proportion of trials for which the animal responded on the right hand side shown as colored dots in blue corresponding to left‐leading ITDs and red to right‐leading ITDs. The fitted psychometric function is shown as a mesh grid. The short black stems seen at some of the data points represent the residuals, that is the difference between the observed responses and those predicted by the fitted psychometric. Rats’ ID and individual time‐intensity‐trading‐ratio (TITR) is indicated in the heading of each plot. D) TITR values for each of the nine tested rats, indicated by differently colored symbols, and the median across all rats (18.7 µs dB^−1^).

To quantify the interaction of ITD and ILD on the binaural perception of our CI rats the data were fitted to a 3D psychometric model. **Figure** **5** shows the fitted models in 3D plots for three representative examples. The fitted psychometric functions were adapted from the cumulative‐gaussian‐with‐lapse functions we introduced in previous publications.^[^ ^17^, ^18^, ^21^, ^22^ ^]^ The model assumes additive interactions between ITD and ILD cues and estimates sensitivity parameters for ITD and ILD, respectively (see Experimental Section for details). Fitting psychometric functions to the data allowed us to estimate TITRs, in µs dB^−1^. Figure 5 shows examples of the fitted psychometric functions as mesh grids for three of the nine CI animals in our study. A strong ILD weighting is indicated by a steep slope of the mesh grid along the ILD‐axis, while a strong ITD weighting is indicated by a steep slope along the ITD‐axis. The examples shown in Figure 5 correspond to the animals with the largest (A), the median (B), and the smallest TITR (C) in our sample. Figure 5D shows the distribution of TITR values across our cohort. The median TITR across all nine CI animals was 18.7 µs dB^−1^, the smallest was 3.9 µs dB^−1^ (rat 9) and the largest 27.1 µs dB^−1^ (rat 1). For illustrations showing psychometric function fits and their residuals for every animal in this study (see Figures S1 and S2, Supporting Information).

## 3 Discussion

This study is the first to investigate the interdependence and interaction between the two binaural cues, ITD and ILD, in a CI stimulated auditory system that is free of the confounding effects of prolonged stimulation with clinical processors. To this end, behavioral experiments were conducted on neonatally deafened, adult CI‐supplied rats, which have already proven to be an excellent model organism for binaural, electric hearing.^[^ ^17^, ^18^, ^19^, ^21^ ^]^ Our experiments revealed that our CI rats combined ITDs and ILDs in an additive manner, with typical TITR values of around 20 µs dB^−1^ or lower. First, this indicates that, even in neonatally deafened, relatively late implanted animals with only modest amounts of experience with electrical stimulation, the manner in which these binaural cues are combined is very similar to what we would expect to see in a normal adult auditory system. Secondly, and perhaps more importantly, the low TITR values we observed indicate that the CI stimulated pathway in a sense “expects” ITDs to be highly informative and useful, and weights them very heavily in localization judgments. Furthermore, although it is sometimes reported that ITD sensitivity tends to decline at high frequencies or pulse rates, we observed these small TITRs at the relatively high stimulation rate (900 pps) commonly used in clinical practice, and while stimulating a relatively high frequency region of the auditory nerve, about half way along the length of the basilar membrane. It therefore does not appear to be the case that only low frequency parts of the auditory pathway are initially set up to expect precise and useful ITD information. Our observations thus clearly contrast sharply with what might be expected based on numerous studies on human CI patients that have documented deficiencies in ITD processing, and while we at present cannot exclude that species differences may play a role here, we nevertheless think it likely that these differences arise from the fact that human patients routinely receive prolonged exposure to random pulse timing ITDs that human patients routinely experience through their clinical devices, while our CI rats do not. We will return to the possibility that plastic changes may drive these differences below, but first we shall try to place our results in their proper context within the previously published literature.

### 3.1 Binaural Cue Sensitivity in Normal Hearing and Deaf Mammals

Acoustic ITD and ILD sensitivity is widespread among mammalian species, and the ITD and ILD thresholds that have been reported for a variety of species are relatively similar: ITD thresholds are typically in the order of a few tens of µs (see **Table** **1**) and ILD thresholds in the order of 1–3 dB (see **Table** **2**). The fairly high degree of similarity in thresholds is perhaps surprising considering that the methods used to determine thresholds in these studies vary considerably.

**Table 1:** Interaural time difference (ITD) threshold across different normal hearing mammals.

Species	ITD threshold	Source
Rats	50 µs	Li et al.^[^ ^22^ ^]^
Cats	30 µs	Wakeford and Robinson^[^ ^23^ ^]^
Guinea pigs	23–45 µs	Greene et al.^[^ ^24^ ^]^
Rabbits	50–60 µs	Ebert, Jr. et al.^[^ ^25^ ^]^
Chinchillas	55 µs	Koka et al.^[^ ^26^ ^]^
Ferrets	40 µs	Keating et al.^[^ ^27^ ^]^
Gerbils	12–96 µs	Tolnai et al.^[^ ^28^ ^]^
Humans	6.9–28 µs	Klumpp and Eady, Zwislocki and Feldman, Mills, Thavam and Dietz^[^ ^29^, ^30^, ^31^, ^32^ ^]^
CI rats	50 µs	Rosskothen‐Kuhl et al., Buck et al.^[^ ^17^, ^18^ ^]^
CI humans	144 µs	Litovsky et al., Laback et al., Noel and Eddington, van Hoesel and Tyler, van Hoesel et al.^[^ ^15^, ^16^, ^33^, ^34^, ^35^ ^]^

**Table 2:** Interaural level difference (ILD) thresholds across different normal hearing mammals.

Species	ILD threshold	Source
Ferrets	1.3 dB	Keating et al.^[^ ^27^ ^]^
Guinea pigs	3 dB	Greene et al.^[^ ^24^ ^]^
Gerbils	0.8 dB	Tolnai et al.^[^ ^28^ ^]^
Rats	3 dB	Wesolek et al.^[^ ^36^ ^]^
Humans	0.5–2.5 dB	Mills et al., Yost and Dye, Grothe et al., Laback et al.^[^ ^12^, ^31^, ^37^, ^38^ ^]^
CI rats	1.7 dB	Buchholz et al.^[^ ^21^ ^]^
CI humans	0.5 to 2.5 dB	Ekloef and Tideholm, Gordon et al., Laback et al., Senn et al.^[^ ^12^, ^13^, ^39^, ^40^ ^]^

Meanwhile, ILD thresholds reported for human CI patients are also similar to those reported for NH humans and animals, in the range of 1–5 dB,^[^ ^12^, ^13^, ^39^, ^40^ ^]^ as is the ILD sensitivity seen in CI rats (median ILD threshold = 1.7 dB^[^ ^21^ ^]^). Bear in mind though, that the encoding of sound levels by a healthy inner ear involves very different physiological mechanisms than the encoding of electrical stimulus amplitudes at the CI / auditory nerve interface. Furthermore, while sound intensities are usually quantified in dB relative to a reference sound pressure, and the dynamic range for sound intensity encoding is very large indeed, CI stimulus intensities are quantified relative to electric current or electric charge, and their physiological dynamic range is quite small. Thus, while it is curious that the NH and CI ILD thresholds reported in the literature turn out to be similar, we have to keep in mind that this may not be a like‐for‐like comparison.

In contrast, acoustic and electric ITDs do measure the same type of quantity in the same units: a stimulus time delay quantified in µs, and acoustic and electric ITDs should be directly comparable. Nevertheless, large differences in ITD sensitivity between NH listeners and CI patients are the norm, and even the best CI users, irrespective of their hearing experience, rarely reach ITD threshold better than 300 µs.^[^ ^15^, ^29^, ^30^, ^32^, ^33^, ^34^, ^35^, ^41^ ^]^ However, considerable variability exists, and Laback et al.^[^ ^16^ ^]^ reported a median ITD threshold of 144 µs. Nevertheless, even star‐performing CI patients are at least one order of magnitude worse than NH humans and the most poorly performing NH non‐human mammals in Table 1.

Human CI patients also contrast sharply with CI rats, which we have repeatedly found capable of achieving excellent ITD sensitivity with thresholds in the tens of microseconds, even at clinical pulse rates up to 900 pps.^[^ ^17^, ^18^, ^19^ ^]^ Of course, one must always be cautious when extrapolating from animal models. Nevertheless, when we consider the cross‐species data we just reviewed as a whole, then the human CI users look like outliers, with NH humans, both NH rats and ND CI rats, and all other species tested achieving thresholds in the range of a few tens of µs. The poor ITD thresholds of human CI patients are exceptional, and they are also unique in that only they are exposed to enormous quantities of random, uninformative and potentially confusing pulse timing ITDs by their clinical CI processing devices.

### 3.2 The Synergy between ITD and ILD Cues

After considering the differences in ITD and ILD sensitivity between normal‐hearing listeners and CI users in isolation, it is worth recalling that these two binaural cues normally interact synergistically. This synergy is advantageous when the cues covary, but in CI patients who receive random pulse timing ITDs through their clinical processors it would become detrimental. Remarkably, however, the data of our CI rats indicates that synergistic interactions between ITD and ILD are nevertheless the “default,” even in an auditory pathway that developed without normal hearing experience and that had only modest experience with electrical hearing.

We found that the synergy could be well described by a simple probit model that assumes an additive interaction between ITD and ILD. That additive interaction captures the “diagonal” color gradients in Figure 3, which are strong across all animals. It also implies that the presentation of congruent ILDs and ITDs makes lateralization easier, leading to a more accurate lateralization performance in electric hearing, and this is clearly confirmed by our data. The nature of this interaction is very much in line with what has been previously described in the normally hearing literature. For example, Sharma et al.^[^ ^42^ ^]^ observed similar synergies in NH subjects, who showed a shorter reaction time when lateralizing clear directional, congruent ITD and ILD cues than when only one of the two cues provided directional information. They therefore concluded that two binaural cues are better than one. In fact, Klingel and Laback^[^ ^43^ ^]^ found a similar effect in CI patients. The linear regression curves fitted to the subjects' lateralization decisions exhibited a significantly steeper slope for the presentation of congruent ITD and ILD cues than for ILDs alone. This already indicated that the addition of a non‐zero ITD to a non‐zero ILD significantly increased lateralization at low pulse rates of 100 and 300 pps. We observed a similar effect in a subset of our CI‐rats tested at a pulse rate of 300 pps, which, as described above, is also visible in the color gradient (Figure S4, Supporting Information). Furthermore, we were able to demonstrate this clear synergetic effect for the first time at a clinical pulse rate of 900 pps and even at a higher pulse rate of 1800 pps, although in this latter case only in a smaller number of rats (Figure S4, Supporting Information).

### 3.3 Weighting of ITD and ILD Cues

As we have seen, ITDs and ILDs interact synergistically, and a model that describes this interaction in terms of a weighted sum is a common way to conceptualize this synergy. A question of great interest is of course what the “appropriate” relative weighting of ITDs and ILDs is or should be. To date, this question has almost exclusively been investigated in the acoustically stimulated auditory system, most likely because the overwhelming majority of bilateral CI patients do not have the requisite ITD sensitivity to make measurements of TITRs in such patients very meaningful. Our discovery that bilaterally CI implanted rats routinely exhibit excellent ITD sensitivity has changed this. In acoustic experiments, TITRs can be examined both electrophysiologically and psychophysically. For example, some studies have used single cell recording in the inferior colliculus of bats to evaluate TITRs. Mean TITR values varied between species from 47 µs dB^−1^ in Mexican free‐tailed bats,^[^ ^44^ ^]^ 30.2±20 µs dB^−1^ in big brown bats,^[^ ^45^ ^]^ and as low as 17.9 µs in pallid bats, for which very similar values were also found on the level of the auditor y cortex (16.7 µs dB^−1[^ ^46^ ^]^). Psychoacoustic studies on normally hearing subjects have identified a number of stimulus parameters that can influence TITRs. For example, Gaskell and Henning,^[^ ^4^ ^]^ found that the TITR for clicks and low frequency pure tones (≈50 µs dB^−1^) was half as large as for high frequency amplitude modulated tones (100 µs dB^−1^), suggesting that the stimulus waveform and frequency have an effect on the TITR. Additionally, adding background noise seems to have a negative influence on the ITD weighting for click and amplitude modulated stimuli. Furthermore, they showed that the TITR decreases with increasing intensity level, i.e., one subject had a TITR of ≈13 µs dB^−1^ for the lowest intensity level of 48 dB, which decreased to less than 5 µs dB^−1^ for the highest intensity level of 93 dB. In addition, the ILD values used for the tests also appear to have an influence, with larger ILD values resulting in larger TITRs.^[^ ^5^ ^]^ In addition to large individual differences, Stecker et al.^[^ ^8^ ^]^ found that the testing procedure can also make a difference. They tested normal hearing subjects with narrowband Gabor clicks in either a “closed‐loop” or an “open‐loop” procedure and found a significantly higher TITR for the “open‐loop” (e.g., 80.2 µs dB^−1^) than for the “closed‐loop” procedure (e.g., 72.4 µs dB^−1^). Furthermore, they examined the influence of the click rate and found a significantly higher TITR for both test procedures with decreasing inter‐click interval from 10 ms (80.2, 72.4 µs dB^−1^) to 2 ms (184.7, 118.7 µs dB^−1^). Another important factor influencing TITRs is the frequency content of the acoustic stimuli used.^[^ ^3^, ^6^, ^9^, ^10^ ^]^ To give just one example, Zerlin et al.^[^ ^3^ ^]^ have shown that the TITR increases with higher stimulus frequencies. At a center frequency of 1000 Hz, the TITR was ≈20 µs dB^−1^, while at 4000 Hz the TITR reached ≈50 µs dB^−1^.

In the framework of this study, using a behavioral testing paradigm, we were able to determine the TITR in an electrically stimulated auditory system. While our main focus was on 900 pps, a pulse rate commonly used clinically, we also collected data from a subset of CI rats at 300 and 1800 pps to investigate potential effects of pulse rate on TITRs (see Figure S3, Supporting Information). At 1800 pps, the data were relatively variable, with mean TITRs of 59 µs dB^−1^, which was higher than at the other pulse rates. This is not surprising given the reduced ITD sensitivity at 1800 pps previously reported for neonatally deafened bilateral CI rats (Buck et al.^[^ ^18^ ^]^). In contrast, at 300 pps, TITRs were considerably lower, ranging between 21–22 µs dB^−1^, making these values very similar to those obtained at 900 pps. At 900 pps, our CI rats reached a median TITRs of 18.7 µs dB^−1^ across all animals (Figure 5B,D) with individual TITRs ranging from 3.9 (Figure 5C) to 27.2 µs dB^−1^ (Figure 5A). Relative to the TITR values from acoustic experiments just reviewed, these values are at the low end, indicating a particularly pronounced influence of ITDs. Due to the already mentioned differences between acoustic and electric hearing, direct comparisons are of course difficult. Nevertheless, the median 18.7 µs dB^−1^ TITR seen in our CI rats implies a surprisingly strong ITD weighting when considered against the background of the many reports of poor ITD sensitivity in CI patients. Our CI rats had even lower TITRs than the 25 µs dB^−1^ TITRs reported for normal hearing human subjects at low frequencies (250 Hz),^[^ ^3^ ^]^ where ITDs are generally thought to be the dominant cue. For our CI rats, a median TITR just below 20 µs dB^−1^ implies that an ITD of only 80 µs should suffice to cancel out an ILD of 4 dB. We have seen in Figure 4A that this is borne out. This underscores that ITD has the potential to be a very powerful cue for CI users, provided that ITDs are delivered in an effective manner to their auditory system.

### 3.4 Consequences of cue Interactions in the Context of Clinical Processor Stimulation

Regrettably, current clinical CI processors are not well adapted to the task of providing microsecond‐accurate temporal information. Clinical stimulation strategies typically deliver pulses at fixed rates of about 1000 pps, which makes it impossible to encode acoustic fine structure with precision. Furthermore, the clocks of the two processors at each ear run independently of each other and independently of incoming acoustic cues. As a consequence, the pulse timing ITDs delivered to the patients are effectively random numbers that could be as large as ±500 µs, large enough to cancel out the effect even of large ILDs. Given that, as we have shown, ITDs can be very powerful cues, it is to be expected that inappropriate, random ITDs delivered by contemporary clinical devices also have great potential to interfere with binaural hearing unless patients learn to become insensitive to this cue. Interestingly, this also appears to be the case at even higher pulse rates, as suggested by our data at 1800 pps. Here we measured a mean TITR of 59 µs dB^−1^, which could still be sufficiently disturbing, given that an offset of ±500 µs in typical clinical processors would correspond to roughly 8 dB.

### 3.5 The Role of Experience and Plasticity in the Weighting of Binaural Cues

The interpretations of our results underscore the importance of auditory training, as plasticity within the CI‐stimulated auditory system may allow users to optimize ITD and ILD integration despite the limitations of current clinical processors. The observed additive effects of ITD and ILD in electric hearing, together with the dependency of TITR on various stimulus parameters and individual differences, suggest that the relative importance of different cues for spatial hearing may be influenced by experience, training, or even short‐term influences such as attention. Examining the experience of our animals, we note that, beyond the common factor of early hearing loss, differences in training prior to the ITD/ILD joint sensitivity measurements described here, were present. Three rats (#2, #6, #9) had a lot of experience lateralizing stimuli based on ITD cues alone^[^ ^19^ ^]^ while the others took part in a study investigating their lateralization performance based on ILD cues alone,^[^ ^21^ ^]^ and it is possible that this may have influenced the TITR values we observed. The TITR in relation to participation in previous studies is presented in Table 1 of the supplements and shows that training with ITDs or ILDs immediately prior to TITR testing does not appear to have a significant effect on the ITD/ILD weighting in bilateral CI rats.

In contrast, numerous studies on normal hearing subjects or bilateral CI patients suggest that training with reinforcement or attention to one specific binaural cue can shift weighting toward that cue. Ignaz et al.^[^ ^47^ ^]^ demonstrated short‐term plastic effects with increasing weighting of one cue in NH humans, when attention was paid to one “to‐be‐adjusted” cue. Klingel et al.^[^ ^48^ ^]^ reinforced the weighting of ITD or ILD cues in NH listeners by presenting additional visual cues and identified that the cue reweighting is frequency dependent in a follow‐up study.^[^ ^10^ ^]^ Additionally, they found that the ability to reweight binaural cues under visual reinforcement applies not only to NH listeners but also to CI listeners, even though it is limited to low stimulation rates of 100 pps. Overall, it remains unclear whether and to what extent experience shapes these effects, and further studies are needed to address this question explicitly.

## 4 Conclusion

Building on the excellent sensitivity to both small ITDs and small ILDs, which can be developed by the early deafened auditory system, this study provides the first behavioral evidence that ITD and ILD cues can either cooperate or compete in lateralization decisions by bilateral CI users, even at clinical stimulation rates. In the absence of clinical stimulation patterns with randomized pulse timing ITDs, the auditory system will tend to combine these cues in a manner that weights ITDs heavily, as evidenced by small TITR values of only 18.7 µs dB^−1^ on average in our CI rats. This naturally high sensitivity to ITD cues suggests that generally poor ITD and generally better ILD sensitivity seen in the overwhelming majority of bilaterally implanted human CI patients may develop as an adaptation post‐implantation, as the patients’ auditory pathway attempts to reduce the potentially severe interference from random pulse timing ITDs, which our experiments have revealed. This interpretation of our finding suggests that reducing the conflict between informative ILDs and uninformative pulse timing ITDs in binaural cochlear implants, perhaps through the wider adoption of fine structure coding strategies, may lead to better binaural hearing outcomes.

## 5 Experimental Section

All procedures involving experimental animals reported here were performed under license approved by the Regierungspräsidium (government council) Freiburg (#35‐9185.81/G‐17/124, #35‐9185.81/G‐22/067). It is confirmed that all of these methods were performed in accordance with the relevant guidelines and regulations and that our study is reported in accordance with the ARRIVE guidelines. A total of nine female Wistar rats were used in this study. All rats underwent neonatal deafening, acoustic and electric auditory brainstem response (ABR/eABR) recordings, bilateral cochlear implantation and behavioral training as described previously^[^ ^17^, ^18^, ^19^, ^21^ ^]^ and briefly below.

### Neonatally Deafening and Cochlear Implantation

All nine rats were neonatally deafened by receiving daily intraperitoneal (i.p.) injections of kanamycin from postnatal day 8–20 inclusively as described previously in Rosskothen‐Kuhl and Illing, Rauch et al., and Rosskothen‐Kuhl et al.^[^ ^49^, ^50^, ^51^, ^52^ ^]^ The ototoxic effect of kanamycin is known to result in the destruction of inner and outer hair cells.^[^ ^53^, ^54^ ^]^ In addition, the number of spiral ganglion neurons is decreased compared to normal‐hearing rats, but is still high enough to ensure electrical excitability of the auditory system. Severe to profound hearing loss (>80 dB) was confirmed by the loss of Preyer's reflex^[^ ^55^ ^]^ and the absence of ABRs to broadband click stimuli. Rats were raised to young adulthood (postnatal week 10–14) and then implanted simultaneously with bilateral cochlear implants (CIs) under ketamine (80 mg kg^−1^) and xylazine (12 mg kg^−1^) anesthesia. Two to three electrodes from either a PEIRA (animal arrays, Cochlear Ltd, Peira, Beerse, Belgium) or a MED‐EL (3‐patch animal arrays, Medical Electronics, Innsbruck, Austria) electrode array were inserted via a cochleostomy over the middle turn (see Table S1, Supporting Information, for details). Note that only the first two tip electrode contacts were used for stimulation in all cases, one as signal electrode, the other as ground. The electrode leads were connected to a Hirose connector which was fixed on the vertex of the animal's cranium with screws and dental acrylic to allow chronic electrical stimulation of the cochlea. The correct function of the CIs was confirmed using eABRs as described previously in Rosskothen‐Kuhl et al. and Rosskothen‐Kuhl et al.^[^ ^51^, ^52^ ^]^

### Electric Stimulation

The electric stimuli used to examine the animals’ eABR and behavioral spatial hearing sensitivity were generated using a Tucker‐Davis Technology (TDT, Alachua, FL) IZ2H programmable constant current stimulator at a sample rate of 48828.125 Hz and delivered directly to the intracochlear electrodes of the head‐mounted percutaneous connector as described above via a custom‐made cable that was connected and disconnected before and after each training session. The cable included a slip‐ring connector and was suspended above the animal's head from a counterbalanced arm, which ensured that the animal could move freely in the behavior box without the cable becoming entangled or impeding the animal's movement. All electrical intracochlear stimulation used biphasic current pulses (duty cycle: 40.96 µs positive, 40.96 µs at zero, 40.96 µs negative), with peak amplitudes of up to 300 µA, depending on eABR thresholds. For behavioral training, all neonatally deafened, cochlear implanted rats were stimulated ≈3.5–4.5 dB above these thresholds. Additionally, the average binaural level (ABL) at which the rats were trained and tested, were confirmed to be reliably detected, but not so high as to cause discomfort (rats will scratch their ears frequently, startle or show other signs of discomfort if stimuli are too intense) by careful observation of the animals’ behavior during spontaneous presentations of test stimuli. For full details on the electric stimuli and stimulation setup see Buck et al., Rosskothen‐Kuhl et al., Schnupp et al., Buchholz et al.^[^ ^17^, ^18^, ^19^, ^21^ ^]^

### Psychoacoustic Training and Testing

After implantation, the rats were trained in a 2AFC sound lateralization task in our custom‐built behavioral setup as described in Buck et al., Rosskothen‐Kuhl et al., Schnupp et al., Buchholz et al.^[^ ^17^, ^18^, ^19^, ^21^ ^]^ The behavioral setup consists of a training cage with three water spouts on one side. A LED would indicate the start of the trial at which point the rats were trained to lick the center spout which would trigger a bilateral CI pulse train. The animals then had to choose between the left and right spout to indicate on which side they heard the stimulus. Correct responses were rewarded with drinking water, incorrect responses triggered negative feedback in the form of a short timeout.

In order to minimize the number of animals used, those included in this study also took part in other studies^[^ ^19^, ^21^ ^]^ as indicated in Table S1 in the Supporting Information. Nevertheless, all CI rats had undergone extensive training with 900 pps pulse trains containing co‐varying ITDs ±{40, 60, 80, 100, 120} µs and ILDs ±{0.5, 1, 2, 3, 4, 5, 6} dB as spatial cues with negative values indicating a leading left ear and positive values a leading right ear. The pulse trains were up to 5 s long, but animals were free to make a choice as soon as they wished after stimulus onset, and the stimulus was terminated as soon as the animal touched one of the response spouts. On average, animals responded within approximately 2 s after stimulus onset.

After the initial training, which lasted between eight and 14 weeks, TITRs of the animals were determined by testing their responses to combinations of ITDs drawn independently from the set of {0, ±60, ±80} µs and ILDs drawn from the set of {0, ±1, ±4} dB. These values were presented to the animals both congruently and incongruently in all possible combinations, except (0 µs, 0 dB). The trials with congruent ITDs and ILDs, which could additionally contain either 0 dB or 0 µs, served as “honesty trials” for which the rats had to respond correctly to receive a reward. For “probe trials” where ITDs and ILDs were presented incongruently, the animals were always rewarded.

The rats were each tested in two sessions per day, five sessions per week. On average, 17 sessions per rat were included in the analysis, with approximately 200 trials per session. Overall, this resulted in an average of 4070 trials collected per animal, of which ≈82% (3356) were honesty trials and ≈18% (714) were probe trials. Only sessions in which the rats scored >75% for honesty trials were included to ensure that the rat listened properly and decided according to its real perception during the probe trials.

For a subset of CI rats (#3, #4, #5, and #8), TITRs were additionally determined at 300 pps and 1800 pps using the same behavioral testing paradigm as described above.

### Data Analysis

To determine the behavioral ITD and ILD sensitivity of our CI rats, the proportion of responses to the right hand side (p_R_) was fit as a function of stimulus ITD and ILD using a psychometric function adapted from the cumulative‐gaussian‐with‐lapse model described in our previous studies,^[^ ^17^, ^18^, ^21^, ^22^ ^]^ under the assumption that ITD and ILD have additive effects. The model takes the form:

(1) $$p_{R}=\Phi \left(\alpha_{\mathrm{ILD}}\cdot \mathrm{ILD}+\alpha_{\mathrm{ITD}}\cdot \mathrm{ITD}+\beta \right)\cdot \left(1-\gamma \right)+\gamma /2+\delta$$

where Φ is the cumulative gaussian distribution function, ITD is the stimulus ITD in µs, ILD is the stimulus ILD in dB, α_ILD_ and α_ITD_ capture the animal's sensitivity to ILD and ITD, and have units 1/dB and 1/µs, respectively, β allows for a possible "ear bias" (if stimuli with 0 ITD and 0 ILD are heard slightly off‐center), *δ* captures a possible "spout bias" (i.e., when an animal guesses, it may have an idiosyncratic preference for one side), and *γ* is a lapse rate parameter that captures the proportion of times the rat makes errors due to inattention or exploratory behavior, irrespective of its ability to discriminate the stimulus. The model was fitted to the observed data using custom code that employed the python library function scipy.optimize.minimize() to implement a gradient descent that maximized the likelihood of the observed data given the set of fitted model parameters. Confirming the suitability of the model for our data, inspection of the residuals, the difference between the observed responses and those predicted by the fitted psychometric, showed no recurring pattern across animals (Figure S2, Supporting Information). TITRs were then computed as the ratio of the sensitivity parameters αITD/αILD. The TITR is defined as the ITD in µs pointing in one direction that is required to offset a 1 dB ILD in the opposite direction, i.e., αILD · 1 ‐ αITD · TITR = 0, which implies TITR = αILD/αITD, or the perception of the stimulus as 0° azimuth.

### Statistical Analysis

Data processing, analysis, and visualization were performed using custom‐written Python scripts with the pandas, seaborn, matplotlib, and scipy packages.

In total, data from nine rats were included in the study, with an average of 4070 trials collected per animal. Of these, approximately 82% (3356 trials) were “honesty trials” and approximately 18% (714 trials) were “probe trials.”

Psychometric functions were fitted using a cumulative‐gaussian‐with‐lapse model under the assumption that interaural time difference (ITD) and interaural level difference (ILD) have additive effects. The model was defined as

(2) $$pR=\Phi \left(\alpha_{\mathrm{ILD}}\cdot \mathrm{ILD}+\alpha_{\mathrm{ITD}}\cdot \mathrm{ITD}+\beta \right)\cdot \left(1-\gamma \right)+\gamma /2+\delta$$

where Φ is the cumulative gaussian distribution function, ITD (µs) and ILD (dB) are the stimulus parameters, αILD and αITD represent sensitivity to ILD and ITD, respectively, β accounts for potential ear bias, δ for spout bias, and γ is the lapse rate parameter capturing random errors due to inattention or exploratory behavior.

Model parameters were estimated by maximizing the likelihood of the observed data using the scipy.optimize.minimize() function implementing gradient descent. Model adequacy was confirmed by visual inspection of residuals, which showed no systematic patterns across animals (Figure S2, Supporting Information). TITRs (µs/dB) were calculated as the ratio of the fitted sensitivity parameters αITD/αILD.

Unless stated otherwise, summary statistics (mean, SEM, median, and 25th–75th percentiles, Figures 4 and 5) refer to values across all animals and were calculated using the stats.sem() function.

## Conflict of Interest

The authors declare no conflict of interest.

## Author Contributions

J.W.H.S., N.R.‐K., and S.B. designed research. N.R.K. and S.B. performed the surgeries. S.B. collected the data. J.W.H.S. and S.B. developed the analysis pipeline. S.B., J.W.H.S., and N.R.K. analyzed the data. S.B., N.R.K., and J.W.H.S. wrote the paper.

## Supporting information



Supporting Information

## Data Availability

All data as well as the analysis code used to generate all the figures and statistical results included in this manuscript are available from the corresponding author on reasonable request. All data generated or analyzed during this study are included in this published article.
