# Supplementary material for: Interactions of Interaural Time and Level Differences in Spatial Hearing with Cochlear Implants
Source: Adv Sci (Weinh). 2025 Nov 14;13(6):e00918. doi: 10.1002/advs.202500918 (PMC12866681; doi:10.1002/advs.202500918)
Supplement: Supplementary file 1 — Supporting Information [file ADVS-13-e00918-s001.docx]

**Supplementary Materials**

| Table S1. Comparing prior study participation and time-intensity trading ratio (TITR) values for each rat, along with information on the number of implanted electrode arrays and electrode manufacturer. | | | | |
| --- | --- | --- | --- | --- |
| Rat # | TITR (µs/dB) | Study prior to cue trading | Electrode manufacturer | Inserted electrodes |
| 1 | 27.2 | Buchholz et al. 2024 (ILDs) [21] | MED-EL | 2 |
| 2 | 25.1 | Schnupp et al. 2025 (ITDs) [19] | MED-EL | 2 |
| 3 | 21.2 | Buchholz et al. 2024 (ILDs) [21] | PEIRA (Cochlear Ltd.) | 2 |
| 4 | 19.9 | Buchholz et al. 2024 (ILDs) [21] | PEIRA (Cochlear Ltd.) | 2 |
| 5 | 18.7 | Buchholz et al. 2024 (ILDs) [21] | PEIRA (Cochlear Ltd.) | 2 |
| 6 | 15.1 | Schnupp et al. 2025 (ITDs) [19] | MED-EL | 3 |
| 7 | 13.0 | Buchholz et al. 2024 (ILDs) [21] | MED-EL | 2 |
| 8 | 12.7 | Buchholz et al. 2024 (ILDs) [21] | PEIRA (Cochlear Ltd.) | 2 |
| 9 | 3.9 | Schnupp et al. 2025 (ITDs) [19] | MED-EL | 3 |

| 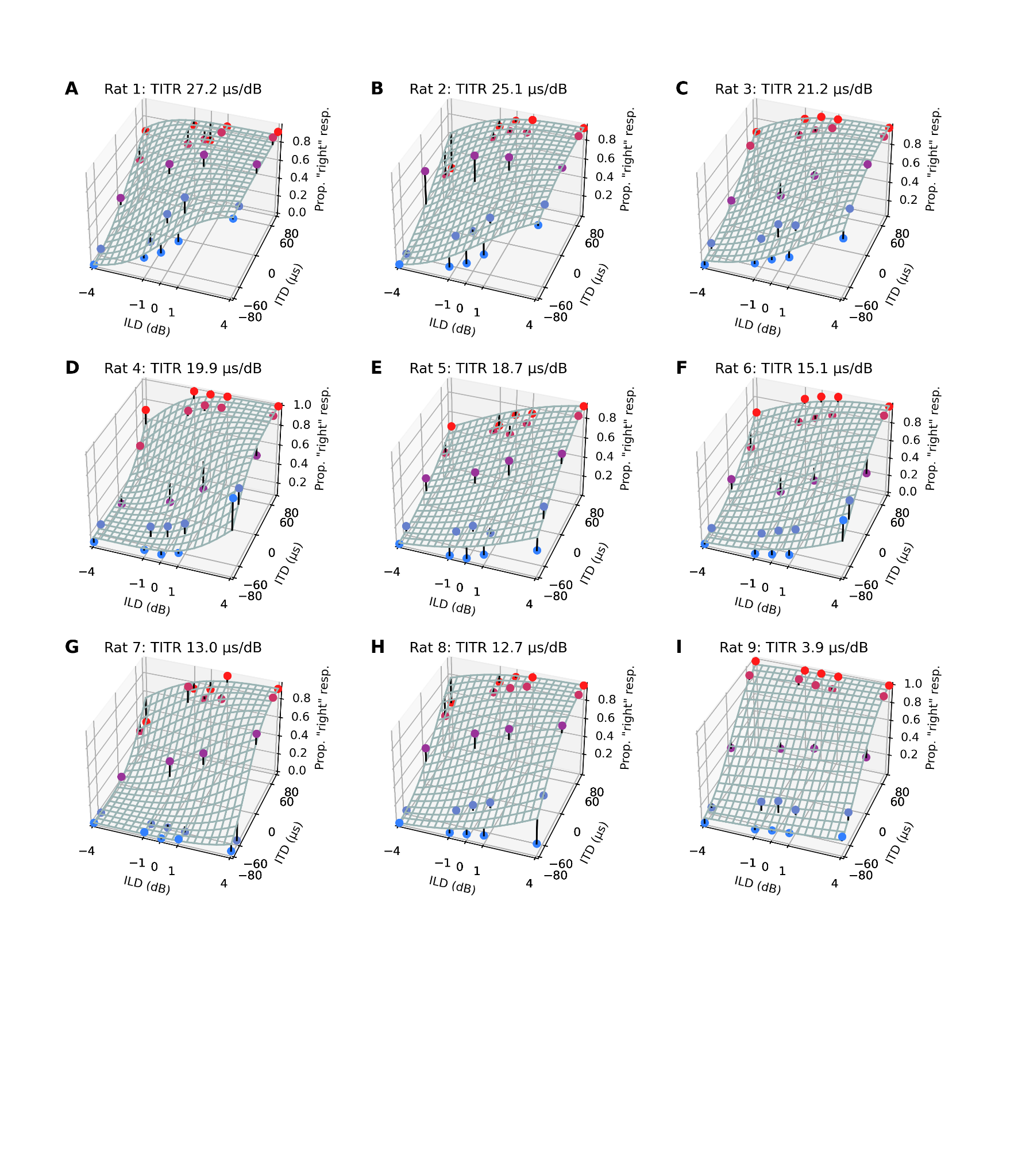 |
| --- |
| Figure S1. Three-dimensional joint interaural time difference (ITD) and interaural level difference (ILD) psychometric functions fitted to the raw data of all nine individual rats. X-axis: ILD values in dB with negative values representing higher intensity on the left ear. Z-axis: ITD values in µs with negative values representing ITDs arriving earlier on the left ear. Y-axis: proportion of trials for which the animal responded on the right hand side shown as colored dots in blue corresponding to left-leading ITDs, respectively. The fitted psychometric function is shown as a mesh grid. The short black stems seen at some of the data points represent the residuals, that is the difference between the observed responses and those predicted by the fitted psychometric. Rats’ ID and individual time-intensity-trading-ratio (TITR) is indicated in the heading of each plot. |

| 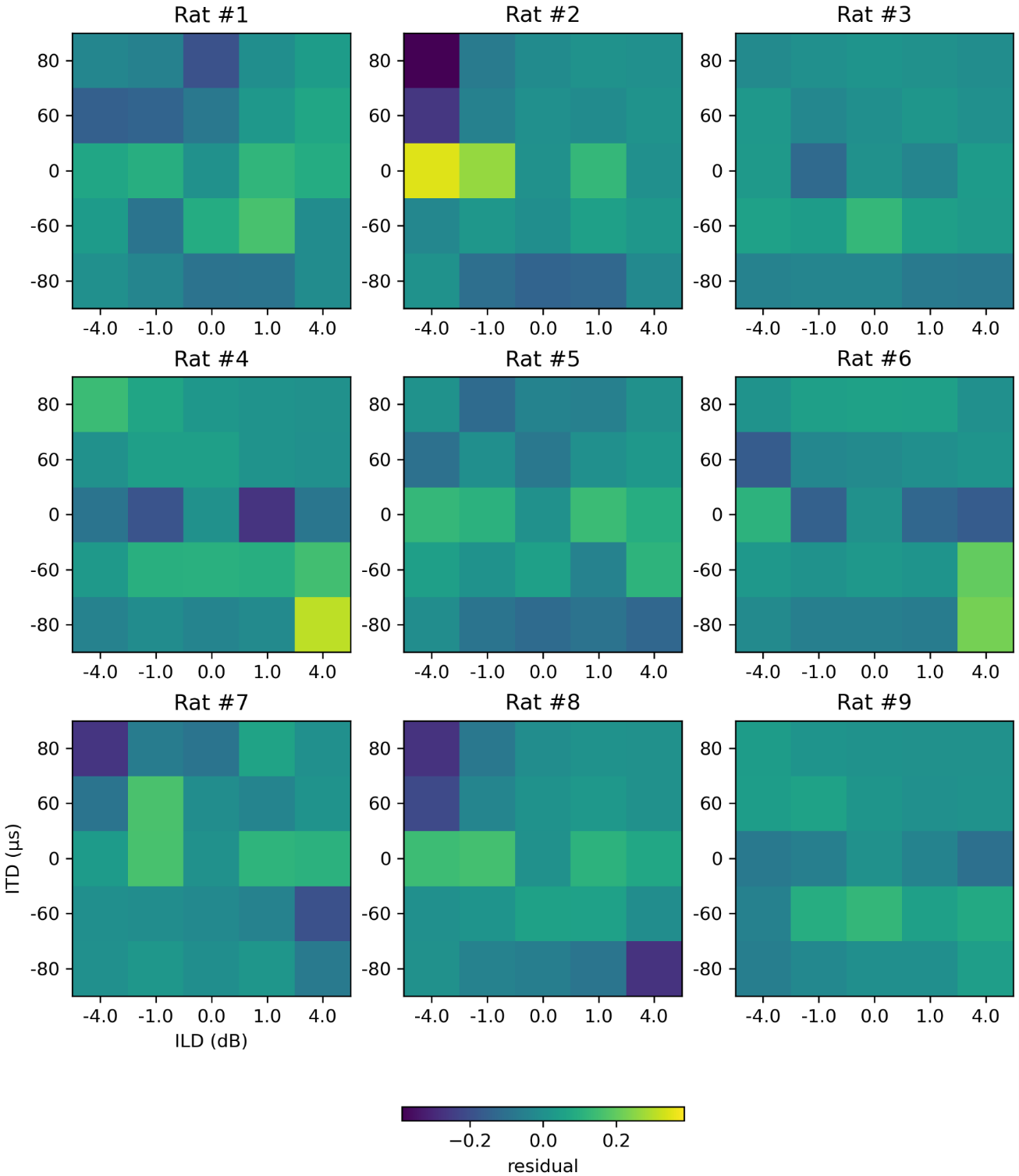 |
| --- |
| Figure S2. Heatmaps showing the residuals, which are the differences between the observed responses of our rats and those predicted by the fitted three dimensional psychometric, also visible as black stems in Fig. S1. The residual of each interaural time difference (ITD, columns) and interaural level difference (ILD, rows) combination is represented in colors according to their values as indicated by the colorbar. |

| 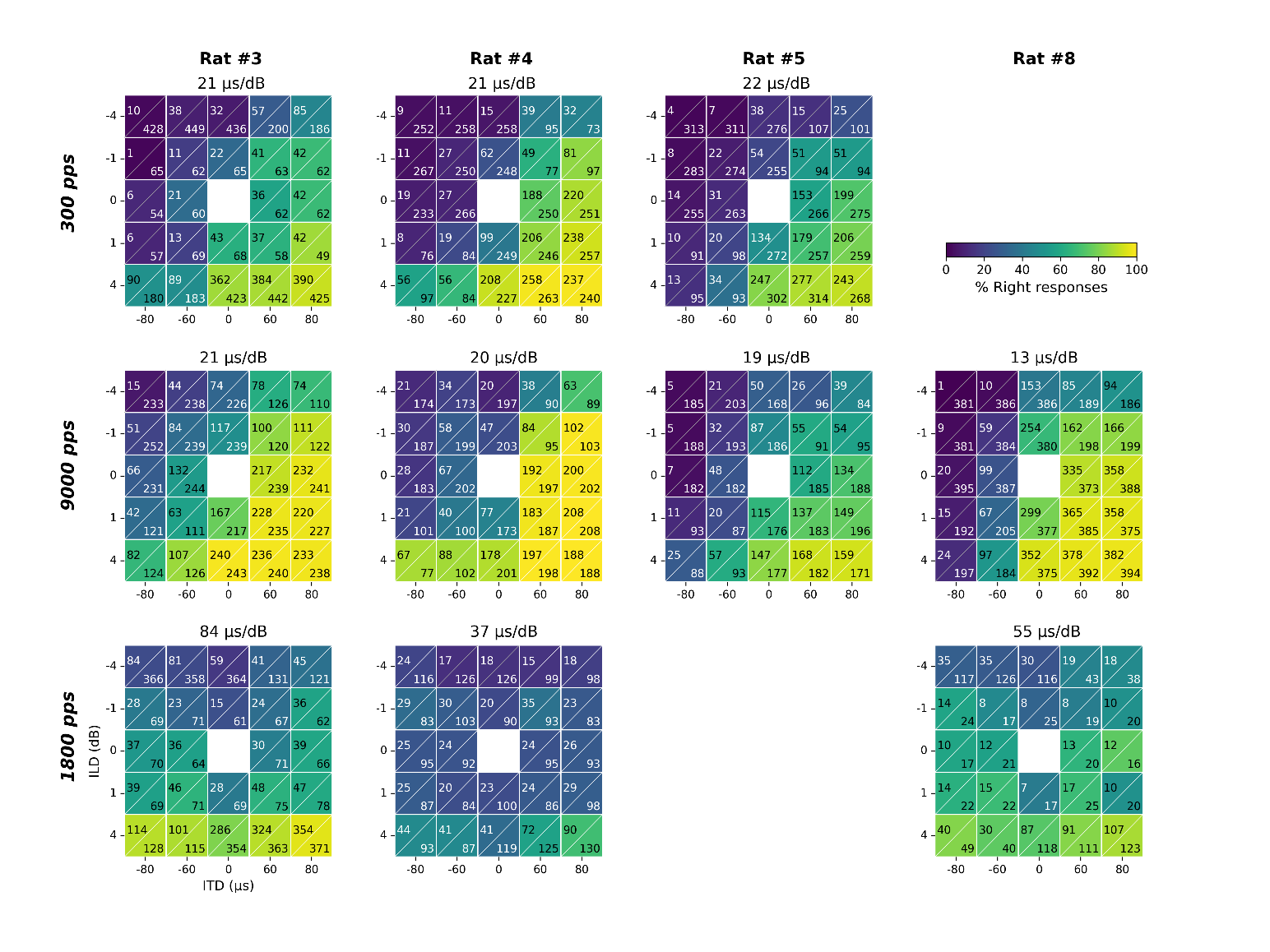 |
| --- |
| Figure S3. Heatmaps showing the proportion of responses to the right hand side for the different ITD (columns) and ILD (rows) combinations for four neonatally deafened cochlear implanted rats (column headings) tested at three different pulse rates (row headings). Each cell shows two numbers; the upper left number indicates the number of responses to the right hand side and the lower, right number gives the total number of presented trials, resulting in the proportion of responses to the right hand side as indicated by the colorbar at the top right. Time-intensity trading ratios (TITR) are shown as heading for each corresponding heatmap. |
